# USE OF PERONEUS LONGUS AS A GRAFT FOR ANTERIOR CRUCIATE LIGAMENT RECONSTRUCTION: A META-ANALYSIS

**DOI:** 10.1590/1413-785220263402e297499

**Published:** 2026-05-22

**Authors:** Yesmin Naji Sola, Joao Vitor de Souza Ribeiro, José Dutra, Helder Rocha da Silva Araújo, Marcelo Rodrigues Torres, Halley Paranhos

**Affiliations:** 1 Centro Estadual de Reabilitacao e Readaptacao Dr. Henrique Santillo, Goiania, GO, Brazil.; 2 Hospital de Urgencias Governador Otavio Lages, Goiania, GO, Brazil.

**Keywords:** Anterior Cruciate Ligament, Anterior Cruciate Ligament Reconstruction, Tendons, Ligamento Cruzado Anterior, Reconstrução do Ligamento Cruzado Anterior, Tendões

## Abstract

To evaluate the advantages and disadvantages of anterior cruciate ligament reconstruction using the long fibular graft, compared to the hamstrings. This is a systematic review with meta-analysis, analyzing articles published in English and Portuguese between 1980 and 2025, in the databases MEDLINE, PUBMED, COCHRANE, and SCIELO. The variables of interest were: IKDC, Lysholm, AOFAS, sample size, and participants’ age. Cochrane's Q test and I2 statistics were used to assess heterogeneity. Significant heterogeneity was identified when p-values were below 0.10, and I2 values were above 25%. Twelve articles were included in the scope of this review. The studies involved 1,212 patients, of whom 72.23% were male, and 18.49% were female. The follow-up time of the patients ranged from six months to five years. No statistically significant differences were found in IKDC and Lysholm of the two samples. There was a small statistical difference in the AOFAS scale. We did not find studies in the literature with sufficient relevance to demonstrate the superiority of long fibular reconstruction as a graft. There are also not enough studies to determine morbidity at the donor site. The use of the long fibular as a graft is promising, but requires caution. ***Level of Evidence I; Systematic Review.***

## RESUMO

Avaliar as vantagens e desvantagens da reconstrução ligamentar do ligamento cruzado anterior utilizando o fibular longo como enxerto, em comparação com os isquiotibiais. Trata-se de uma revisão sistemática com metanálise, com análise de artigos publicados nas línguas inglesa e portuguesa entre 1980 e 2025, nas bases de dados: MEDLINE, PUBMED, COCHRANE E SCIELO. As variáveis de interesse foram: IKDC, Lysholm, AOFAS, tamanho da amostra e idade dos participantes. O teste Q de Cochrane e as estatísticas I^2^ foram utilizadas para avaliar a heterogeneidade. Determinou-se heterogeneidade significante para valores de p abaixo de 0,10 e valores de I^2^ acima de 25%. Fizeram parte do escopo dessa revisão 12 artigos. Os estudos envolveram 1212 pacientes, 72,23% do sexo masculino e 18,49%, do sexo feminino. O tempo de seguimento dos pacientes variou de seis meses a cinco anos. Não foram encontradas diferenças estatísticas significativas em IKDC e Lysholm das duas amostras. Houve uma pequena diferença estatística na escala AOFAS. Não encontramos na literatura estudos com relevância o suficiente que consigam comprovar a superioridade da reconstrução do fibular longo como enxerto. Também não há estudos o suficiente para determinarmos morbidade no sítio doador. O uso de fibular longo como enxerto é promissor, mas requer cautela. ***Nível de Evidência I; Revisão Sistemática.***

## INTRODUCTION

The anterior cruciate ligament (ACL) is the primary static stabilizer of anterior knee stability; it is the second most commonly injured structure in the knee, after the menisci, with an annual incidence of approximately 100,000 to 200,000 cases in the United States alone.^1^

The leading cause of injuries remains sports activities. An ACL tear can result in permanent and severe complications such as knee instability, cartilage damage, and meniscal damage, which can lead to osteoarthritis and negatively impact the individual's quality of life.^2^

The gold standard treatment is arthroscopic reconstruction of the anterior cruciate ligament using an autograft. Common choices for grafting include the patellar tendon, quadriceps tendon, and hamstring tendons. However, some postoperative drawbacks include anterior knee pain and weakness of the extensor mechanism. The hamstring tendon (HT) is currently the most commonly used autograft for ACL reconstruction.^3^

Currently, the use of the long fibular tendon has gained prominence due to its ability to reduce residual morbidity at the donor site^3^ and because it shows no difference in tensile strength and yields better functional scores compared to the hamstring tendon.^2^ The choice of graft is patient-centered, considering anatomical variations, patient preference, reduction of morbidity, and appropriate rehabilitation. Therefore, expanding the range of options makes ACL reconstruction more effective.

The main objective of this study is to present satisfactory clinical and functional outcomes following the use of the long fibular tendon in arthroscopic ACL reconstruction, compared with the most commonly used graft, the hamstring, through a systematic review and meta-analysis of randomized clinical trials.

## METHODOLOGY

The formulation of the research question and search strategy for this article was based on the PICO model (*Population, Intervention, Comparison, Outcome)*, which is widely used in evidence-based practice methodology and recommended for conducting systematic reviews. The PRISMA model was used as a reference for the article selection flowchart.

### Search strategy

The study included articles published between 1980 and 2025 in English and Portuguese that described the use of the long fibular in anterior cruciate ligament reconstruction, using the following databases as references: MEDLINE, PUBMED, COCHRANE E SCIELO. For the initial search, the following search terms were used: *"anterior cruciate ligament*" combined with *"*rec*onstruction*" and "peroneus *longus*."

The articles were selected by two independent reviewers based on the title and abstract. The potentially eligible articles were read in their entirety. Afterward, the reviewers also reviewed the reference lists of all eligible articles to identify additional references for this study.

### Eligibility criteria

The inclusion criteria were: (1) population (adults, aged 16 years and older); (2) intervention (ACL reconstruction using the peroneus longus tendon compared with the flexor tendons); (3) outcomes of interest (functionality, quality of life); (4) articles published in the last 45 years—in English or Portuguese; (5) randomized clinical trials, prospective observational studies, retrospective observational studies, cross-sectional studies, case-control studies; (6) studies with full-text versions available in the searched databases; (7) primary and isolated ACL reconstruction.

### Exclusion criteria

The exclusion criteria were: (1) studies involving participants under the age of 16; (2) studies involving patients with multi-ligament injuries; (3) studies that did not compare the use of flexor tendons and the long fibular tendon; (4) other types of articles, such as case reports, biomechanical studies, surgical techniques, and others not selected based on the inclusion criteria.

### Data extraction

After completing the previous steps, a reviewer extracted the following data from each article: year of publication, sample characteristics (sample size, population, age, gender), surgical technique, follow-up period, and outcomes (functionality and quality of life).

The variables of interest were entered into a spreadsheet by one of the authors using Excel (Microsoft Corp., United States). The relevant data were analyzed using descriptive statistics.

The risk of bias assessment for randomized trials was conducted independently by two authors using the Risk of Bias for Randomized Trials (ROB-2) tool, in accordance with Cochrane guidelines.

The results were compared using the standardized mean difference (SMD), and binary outcomes were assessed using risk ratios (RR), with their respective 95% confidence intervals (CI). The DerSimonian and Laird method was used to conduct the random-effects meta-analysis. Statistical analyses were performed using Review Manager 5.4 (The Cochrane Collaboration, London, United Kingdom).

The Cochrane Q-test and I^2^ statistics were used to assess heterogeneity. Significant heterogeneity was identified when p-values were below 0.10, and I^2^ values were above 25%. The interpretation of heterogeneity measures followed the guidelines provided in the Cochrane Handbook for Systematic Reviews of Interventions.

## RESULTS

Based on the search terms used and the publication date specified by the authors, a total of 120 articles, including 96 from PubMed, 8 from MEDLINE, 3 from SCIELO, and 13 from Cochrane.

**Figure 1:**
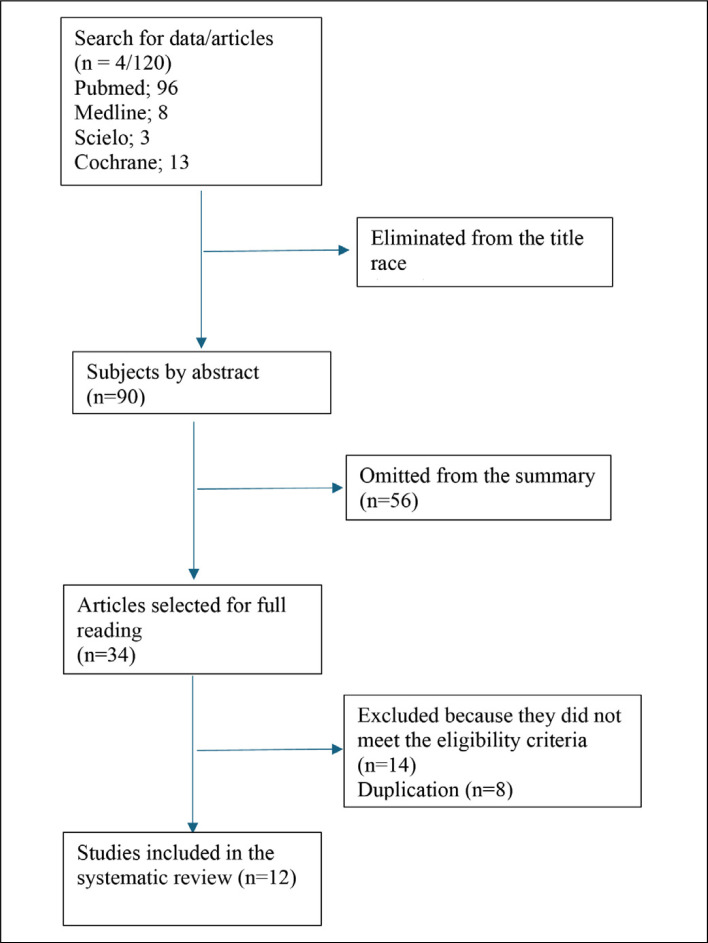
Flowchart.

After reviewing the titles and abstracts, 86 articles were excluded based on the eligibility criteria. Among the most common reasons for exclusion are: studies that did not compare grafts, biomechanical studies, and studies that did not include the desired outcomes. Articles for which the full text was not available were also excluded. The selected studies (34) were screened for duplication, resulting in 8 articles. Subsequently, the articles were read in full, and 10 were excluded because they lacked data relevant to this review. Following the selection process and the application of the eligibility criteria, 12 articles were included in this systematic review. The PRISMA flowchart was used to aid understanding (Figure 1).

The data extracted for the analysis were then summarized in a table for easier visualization, including the following variables: year of publication, type of study, sample size and characteristics, surgical technique, patient follow-up period, and outcomes measured using specific scales (IKDC, Lysholm, AOFAS, FADI) (Table 1).

**Table 1:** Data analysis.

Author / Year	Type of Study	Sample size	Genre	Age	Surgical technique	Follow-up period
Agarwal et al. (2023)^4^	Prospective cohort study	194 patients Long fibular group: 98 Flexor muscle group: 96	Men's: Women's 125: 69	Long fibular: 28± 4.91 Flexors: 27.50 ± 4.06	Femoral fixation with an endobutton Tibial fixation using a bio-absorbable interference screw	12 months
Dwidmuthe et al. (2024)^5^	Prospective randomized study	36 patients Long fibular group: 18 Flexor muscle group: 18	Men's: 29 Women's: 7	29.27±7.87	Femoral fixation with a closed-loop endobutton Tibial fixation using a bio-absorbable interference screw	6 months
Saeed, et. al (2023)^1^	Prospective cohort study	158 patients Long fibular group: 85 Flexor muscle group: 73	Men's: 138 Women's: 20	29.55 ± 6.40	Femoral fixation with an endobutton Tibial fixation using a bio-absorbable interference screw	24 months
Asif et al. (2024)^6^	Randomized controlled clinical trial	120 patients Long fibular group: 60 Flexor muscle group: 60	Men's: Women's 112: 8	Long fibular: 24.4 ± 9.54 Flexors: 24.73 ± 8.18	Femoral fixation using an endobutton with a fixed loop or an adjustable loop Tibial fixation using an interference screw, tibial post screw, or tibial suture disk	24 months
Gok et al. (2024)^7^	Comparative cohort study	106 patients Long fibular group: 52 Flexor muscle group: 54	Men's: 93 Women's: 13	Long fibular: 28 ± 6.2 Flexors: 28.9 ± 6.1	Femoral fixation with an endobutton Tibial fixation using a bio-absorbable interference screw	18 months
Shi et al. (2018)^8^	Observational study	38 patients Long fibular group: 18 Flexor muscle group: 20	Not specified	Long fibular: 42 Flexors: 40	Femoral fixation with an endobutton Tibial fixation prior to 2008 using the transtibial drilling technique and, thereafter, drilling of separate anteromedial femoral tunnels	24 months
Rhatomy et al. (2019)^9^	Prospective observational study	52 patients Long fibular group: 24 Flexor muscle group: 28	Men's: 44 Women's: 8	Long fibula: 23.4 ± 8.1 Flexors: 26.4 ± 8.6	Femoral fixation with an endobutton Tibial fixation using a bio-absorbable interference screw	12 months
Butt et al. (2024)^3^	Prospective randomized study	60 patients Long fibular group: 30 Flexor muscle group: 30	Men's: 59 Women's: 1	Long fibular: 29.2 ± 5 Flexors: 27.7 ± 4.1	Femoral fixation with ACL Tight rope Tibial fixation using a bio-absorbable interference screw	5 years
Keyhani et al. (2022)^2^	Cross-sectional study	130 patients Long fibular group: 65 Flexor muscle group: 65	Men's: 119 Women's: 11	Long fibular: 29.8 ±7.5 Flexors: 27.60± 8.1	Femoral fixation with ACL Tight rope Tibial fixation using a bio-absorbable interference screw	2 years
Mingguang et al. (2018)^10^	Prospective randomized study	124 patients Long fibular group: 62 Flexor muscle group: 62	Men's: 65 Women's: 59	Long fibular: 29.1 ± 6.5 Flexors: 27.9 ± 6.7	Femoral fixation with ACL Tight rope Tibial fixation with ACL Tight rope	2 years
Ahmed et al. (2022)^11^	Prospective comparative study	75 patients Long fibular group: 25 Flexor muscle group: 25 Group quadriceps: 25	Men's: 57 Women's: 18	Long fibular: 33.3 ± 6.4 Flexors: 31.5 ± 3.9	Femoral fixation with ACL Tight Rope and interference screws Tibial fixation using a bio-absorbable interference screw	12 months
Vijay et al. (2022)^12^	Prospective randomized study	45 patients Long fibular group: 23 Flexor muscle group: 22	Men's: 35 Women's: 10	Long fibular: 33.57 ± 9.54 Flexors: 31.82 ± 9.62	Not specified	12 months

The studies involved a total of 1,212 patients, of whom 72.23% were male (876) and 18.49% were female (224). Shi et al.^8^ did not report this. Among the grafts used, 560 were taken from the peroneus longus and 553 from the flexor muscles. The follow-up period for patients ranged from six months^5^ to five years.^3^

**Figure 2:**
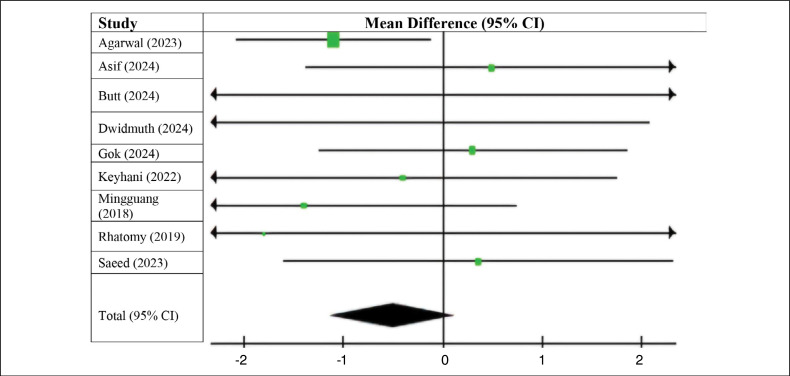
Preoperative IKDC.

The graphs show that patients who underwent ACL reconstruction using an autologous long fibular graft had higher preoperative IKDC scores compared to those who underwent reconstruction using a flexor tendon graft (MD −0.50; 95% CI −1.13 to 0.13; p=0.48; I^2^=0%), however, there was no statistically significant difference (p > 0.05). (Figure 2)

**Figure 3:**
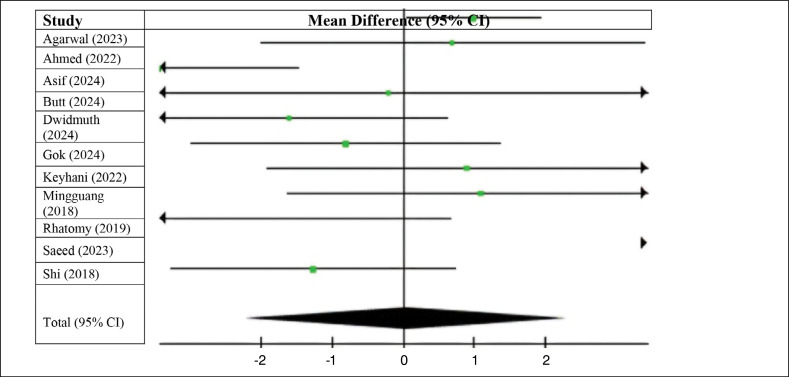
Postoperative IKDC.

**Figure 4:**
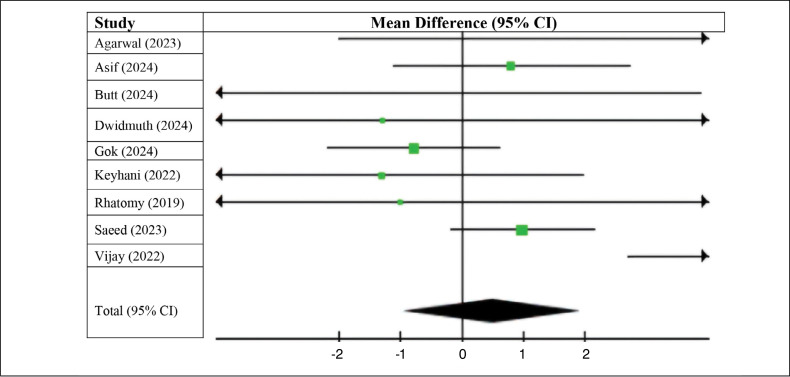
Preoperative Lysholm score.

When examining postoperative IKDC scores, we observed scores that slightly favored the use of flexors (MD 0.05; 95% CI −2.21 to 2.31; p < 0.00001; I^2^ = 93%). Nevertheless, the difference was not significant, as the confidence interval has a value of 0. (Figure 3) The long fibular group had lower preoperative Lysholm scores (MD 0.50; 95% CI −0.95 to 1.94; p = 0.04; I^2^ = 95%); however, these differences were also not statistically significant (Figure 4).

**Figure 5:**
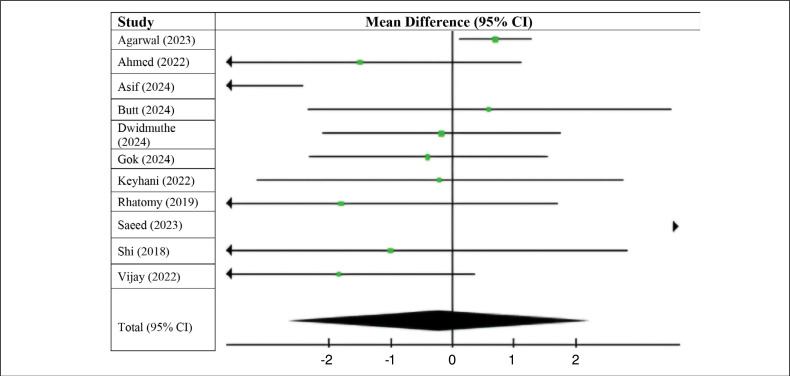
Postoperative Lysholm score.

On the other hand, the long fibular group had higher postoperative Lysholm scores (MD −0.21; 95% CI −2.67 to 2.25; p < 0.00001; I^2^ = 96%) compared to the group that used flexors (Figure 5). Nevertheless, our confidence interval includes 0, so there is no statistically significant difference either.

**Figure 6:**
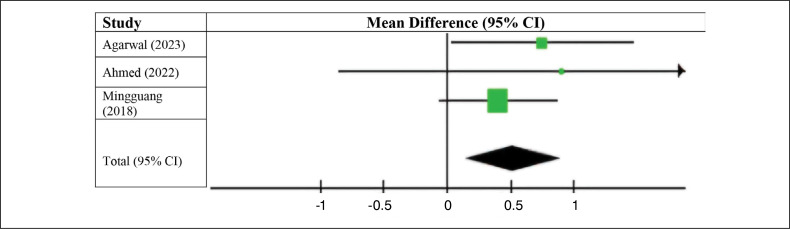
Postoperative AOFAS score.

Patients who underwent ACL reconstruction surgery using autologous long fibular grafts had lower postoperative AOFAS scores (MD 0.52; 95% CI −0.14 to 0.90; p < 0.07; I^2^ = 0%) (Figure 6) compared with those who received autologous flexor grafts. This difference was considered statistically significant.

**Figure 7:**
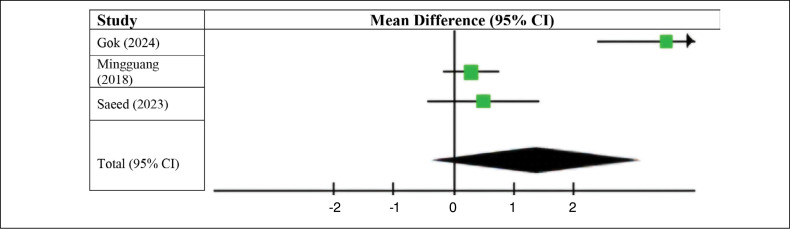
AOFAS score with the use of the peroneus longus tendon.

We also analyzed the pre- and postoperative AOFAS scores in patients in the group who underwent ACL reconstruction surgery using the long fibular. The results favored the preoperative group (MD 1.39, 95% CI −0.35 to 3.12, p < 0.00001; I^2^ = 93%). As with previous results, we did not observe a statistically significant difference (Figure 7).

## DISCUSSION

The strength and stiffness of the graft are important factors to consider when choosing the type of graft and the reconstruction technique. It is widely accepted that an autologous hamstring tendon graft is a reliable option for ACL reconstruction; however, the use of alternative grafts has been increasing significantly.

The aim of this meta-analysis was to compare the clinical outcomes of anterior cruciate ligament (ACL) reconstruction using autografts from the long fibular tendon versus flexor tendons. Overall, both types of graft yielded similar results on the main functional scores (IKDC, Lysholm, AOFAS, FADI), with differences that were, for the most part, not statistically significant. This similarity underscores the potential of the long fibular tendon as a viable alternative to the grafts traditionally used most often, as suggested by Shi et al.^8^ and corroborated by Ahmed et al.^11^

Regarding the preoperative IKDC score, patients in the group using the long fibular tendon had slightly higher scores, although this difference was not statistically significant. This finding may reflect a selection bias in some studies, since patients with different functional characteristics may have been allocated differently across groups, as noted by Keyhani et al.^2^ In the postoperative period, there was a slight advantage for the flexor group, but again without statistical significance, which is consistent with the results of Rhatomy et al.^9^ and Gok et al.^7^ who found no significant differences in functional recovery among patients operated on with different types of grafts.

Regarding the Lysholm score, the long fibular group had lower preoperative scores and slightly higher postoperative scores, although the difference was not statistically significant. These results support the notion that the long fibular muscle provides functional stability comparable to that of the flexor muscles, as also noted by Dwidmuthe et al.^5^ and Keyhani et al.^2^, especially when used in conjunction with appropriate fixation and rehabilitation techniques.

On the other hand, the postoperative AOFAS score was the only outcome measure that showed a statistically significant difference, favoring the flexor group. This finding, also noted by Saeed et al.^1^, suggests that, although functionally similar in most respects, the flexor tendon graft may still offer a slight advantage in terms of pain and distal functional capacity of the operated limb, especially in the medium term.

It is important to note, however, that the high heterogeneity observed in several analyses (I^2^ above 90%) limits the generalizability of the findings. This variability can be attributed to methodological differences among the studies, including follow-up duration (ranging from 6 months to 5 years), surgical techniques, rehabilitation protocols, and clinical assessment criteria.

In addition, several methodological limitations are worth noting. The lack of blinding of evaluators in some studies, inconsistencies in patient inclusion criteria, and the absence of complete data in certain articles may have affected the robustness of the statistical synthesis. The still-limited number of high-quality studies—particularly randomized clinical trials with large sample sizes—underscores the need for more standardized future research.

Despite these limitations, the findings of this meta-analysis provide valuable insights for clinical practice. The long fibular tendon has proven to be a valid alternative to flexor tendons in ACL reconstruction, particularly in cases where the use of flexor tendons is contraindicated, when graft diameters are inadequate, or when the flexor tendons have been previously used as autografts. It is up to surgeons to consider each patient's individual characteristics, as well as the team's experience and available resources, when selecting the most appropriate graft.

## CONCLUSION

After analyzing 12 studies comparing the use of the peroneus longus tendon with the hamstring tendons, it is evident that autologous peroneus longus tendons are promising, yielding functional outcomes similar to those of traditional tendons.

However, a higher incidence of morbidity is observed at the donor site compared to the contralateral ankle. It should be noted that the number of studies with Level 1 evidence regarding ankle morbidity is limited; therefore, the use of the long fibular should be approached with caution. It can be concluded that autologous long fibular grafts do indeed yield satisfactory results in the knee; however, their implications for the ankle and foot, as well as additional studies with larger sample sizes and long-term follow-up, may provide further insight into their use.

## Data Availability

The contents underlying the research are available in the manuscript.
